# Prostatic artery embolization with glue for benign prostatic hyperplasia in elderly patients: three-year results

**DOI:** 10.1007/s11547-025-02136-2

**Published:** 2025-11-18

**Authors:** Antonio Vizzuso, Maria Vittoria Bazzocchi, Mara Bacchiani, Giorgia Musacchia, Antonio Spina, Eugenia Fragalà, Giovanna Venturi, Enrico Petrella, Roberta Gunelli, Emanuela Giampalma, Matteo Renzulli

**Affiliations:** 1 Radiology Unit, Morgagni-Pierantoni Hospital, AUSL Romagna, 47121 Forlì, Italy; 2 Department of Urology, University of Florence, Florence, Italy; 3 Urology Unit, Morgagni-Pierantoni Hospital, AUSL Romagna, 47121 Forlì, Italy; 4 Medical Physics and Clinical Engineering Operative Unit, Morgagni-Pierantoni Hospital, AUSL Romagna, 47121 Forlì, Italy; 5 Department of Medical and Surgical Sciences, University of Bologna, 40100 Bologna, Italy

**Keywords:** Prostatic artery embolization, Interventional radiology, Glue, Lower urinary tract symptoms, Indwelling catheter

## Abstract

**Purpose:**

To evaluate the clinical efficacy and safety of prostatic artery embolization (PAE) using glue (n-butyl cyanoacrylate, NBCA) in patients with benign prostatic hyperplasia (BPH) presenting with lower urinary tract symptoms (LUTS) due to obstruction or chronic urinary retention managed with an indwelling catheter (IUC).

**Material and Methods:**

A total of 101 patients (median age 79 years) were included, of whom 67 had LUTS and 34 had an IUC. All were treated with PAE with glue between 2021 and 2024. Clinical success was defined as either a ≥ 25% reduction in the International Prostate Symptom Score (IPSS) and a ≥ 3-point improvement in the quality of life (QoL) score or stable catheter removal.

**Results:**

Technical success was achieved in 100% of cases, with bilateral embolization in 93% of patients. Among symptomatic patients, clinical success was observed in 92.5%, with a reduction in IPSS from 25.3 ± 8.1 to 15.3 ± 7.8 and in QoL from 4.5 ± 1.1 to 2.3 ± 1.4 at 36 months (*p* < 0.001). Mean prostate volume decreased by 37.6%. In patients with an IUC, 73.5% resumed spontaneous voiding within an average of 29 days. All complications (13.9%) were minor and managed conservatively.

**Conclusion:**

Embolization with acrylic glue is a safe and effective minimally invasive alternative to surgery for BPH, with sustained long-term results. It is particularly suitable for elderly patients with comorbidities or those unfit for surgery presenting with LUTS or chronic urinary retention.

## Introduction

In the last years, prostatic artery embolization (PAE) has emerged as a minimally invasive treatment for lower urinary tract symptoms (LUTS) secondary to benign prostatic hyperplasia (BPH) [1, 2]. Transurethral resection of the prostate (TURP) remains the standard treatment for medically refractory LUTS [1]. However, PAE has emerged as an alternative, with studies demonstrating non-inferiority to TURP in terms of clinical outcomes and a more favorable adverse event profile [1–3]. Notably, PAE is associated with significant short-term improvements in International Prostate Symptom Score (IPSS) and quality of life (QoL), with minimal impact on erectile function [1]. Despite these advantages, symptom relief following PAE tends to be less durable, with recurrence rates of up to 23% at six years when using microspheres [2]. This limited long-term efficacy has contributed to the lack of consensus in international guidelines regarding its routine clinical application. The European Association of Urology (EAU) guidelines state that PAE may be offered to patients with moderate to severe LUTS who are unfit for or decline surgery. However, patients should be aware of the risk of potentially suboptimal urodynamic outcomes [4, 5]. The American Urological Association (AUA) recommends PAE only within the context of clinical trials or as an investigational procedure, citing insufficient long-term data [4, 6].

Originally developed using microspheres with the standard ‘PerFecTED’ technique, PAE has evolved significantly over time [6]. To improve long-term outcomes and reduce complications, various embolic agents and devices have been tested, including drug-eluting particles [8, 9], coils [10], and balloon-occlusion microcatheters [11, 12]. Among these, the liquid embolic agent n-butyl cyanoacrylate (NBCA) glue has recently attracted particular interest [13]. When mixed with ethiodized oil, NBCA provides excellent fluoroscopic visibility and controlled distal penetration, minimizing non-target embolization. Its rapid polymerization ensures immediate occlusion and shortens the embolization phase, reducing radiation exposure [-14–15]. Several studies indicate that glue-based embolization is non-inferior to microparticle embolization in terms of efficacy, achieving comparable improvements in IPSS, QoL, prostate volume (PV) reduction, and peak flow (Q_max_) increment [14, 16–18]. Furthermore, PAE performed with glue has been associated with successful removal of long-term indwelling urinary catheters (IUC), indicating potential efficacy in selected patients with chronic urinary retention (CUR) [19].

Although concerns have historically been raised regarding the risk of ischemic complications and non-target embolization with glue, current evidence supports that glue-based PAE does not confer a higher complication rate compared to microspheres. Reported adverse events have been predominantly minor and self-limiting [14, 16–18]. These properties, combined with the ability to overcome anatomical challenges, such as tortuous or small-caliber prostatic arteries that are difficult to catheterize, confer a distinct advantage over traditional microparticles [13].

Despite these promising results, most studies offer only short-term follow-up [14, 16–18]. The longest published follow-up of NBCA-based PAE reported sustained improvements in IPSS, QoL, prostate-specific antigen (PSA) levels, and PV over a 6-month period, yet long-term outcomes remain to be fully elucidated [17].

The aim of the present study is to evaluate the performance of NBCA-based PAE in an elderly patient population with a long-term follow-up.

## Materials and methods

### Study design

This retrospective observational study was conducted at our institution in accordance with the Declaration of Helsinki. Ethical approval was waived due to the use of anonymized clinical data. All patients were informed about the use of NBCA at the time of the PAE procedure. A prospectively maintained database was retrospectively reviewed to identify consecutive patients with LUTS or CUR with IUC due to BPH treated with NBCA-PAE between 2021 and 2024.

Eligibility criteria for patients with LUTS included the presence of moderate to severe symptoms (IPSS > 7 and QoL > 2) persisting for at least six months, with either inadequate response or intolerance to medical therapy with α1-adrenergic receptor antagonists and/or 5α-reductase inhibitors, who declined surgical intervention or had medical contraindications to surgery. A PV greater than 40 mL on magnetic resonance (MR) was required for inclusion.

Exclusion criteria included active urinary tract infection, a PI-RADS score ≥ 3 on pre-procedural multiparametric MR imaging, histologically confirmed prostate cancer, or advanced renal impairment. Clinical success was defined as ≥ 25% IPSS reduction and QoL ≤ 3 for patients with LUTS [20] and removal of IUC in men with CUR prior to PAE [2, 17].

### PAE procedure

All procedures were performed by interventional radiologists, each with a minimum of five years of experience in endovascular procedures, using local anesthesia (without conscious sedation) and the right transfemoral approach. In cases where femoral access was not feasible, an alternative right radial artery approach was performed. In patients without an IUC, a temporary bladder catheter was routinely inserted prior to embolization. The balloon was inflated using a mixture composed of one-third iodinated contrast medium and two-thirds normal saline. Once identified and entered into the prostatic artery, Cone-Beam CT (CBCT) is systematically performed to assess the perfusion of the central gland (CG) and to identify potential anatomical variants, particularly Type 3 vascular anatomy, which is characterized by two independent, non-communicating arterial pedicles supplying separate compartments of the CG and therefore requires embolization of both feeders to ensure complete treatment [21]. If the entire hemi-gland is adequately opacified, embolization is performed, and no post-embolization digital subtraction angiography (DSA) control is carried out. Conversely, if CBCT reveals incomplete perfusion of the CG, a new DSA evaluation was carried out to identify and selectively catheterize additional feeding branches.

**Fig. 1:**
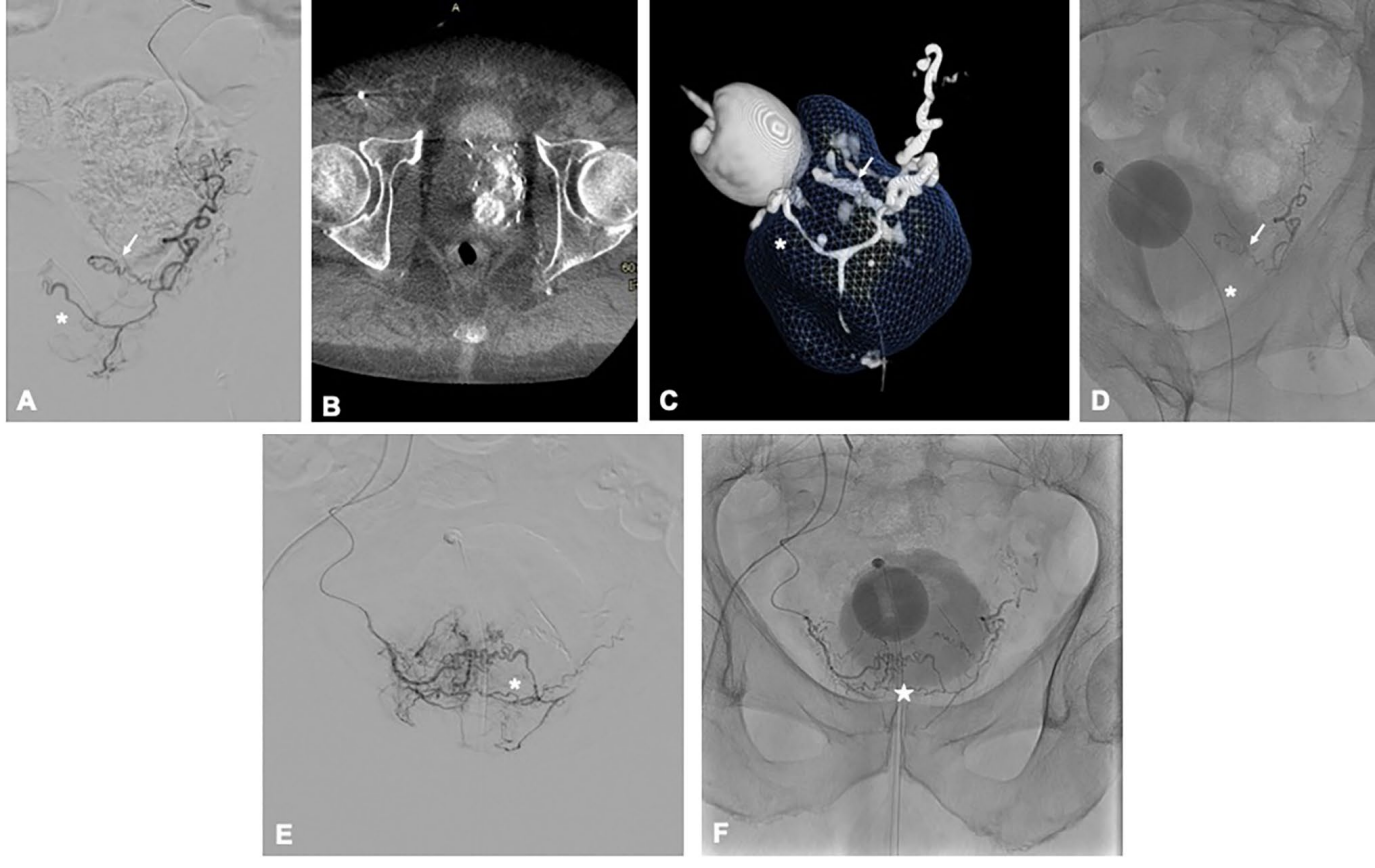
PAE with NBCA in an 80-year-old patient with an IPSS of 23. **A** Left oblique angiographic image shows the left prostatic artery dividing into a posterior–cranial branch directed to the third lobe (arrow) and an anterior–caudal branch (asterisk). **B** Axial Cone-Beam CT image acquired after contrast injection (1 mL/s flow rate, 4 mL total volume, 6-s acquisition delay) demonstrates opacification of the entire left prostatic hemigland. **C** 3D reconstruction of the prostate (volume: 74 mL) shows the intraparenchymal branching (arrow and asterisk) of the left prostatic artery. **D** Post-embolization angiogram shows glue–oil cast in the posterior branch (arrow) but not in the anterior–caudal branch (asterisk). **E** Angiogram of the right prostatic artery reveals perfusion of the right hemigland and the untreated portion of the left hemigland (asterisk). **F** Final angiographic image demonstrates distribution of the glue–oil cast within the parenchymal branches of the prostate (star) and along both right and left prostatic arteries

After selective catheterization of the prostatic artery, the nitroglycerin was injected and the 2.5 Fr microcatheter (RenegadeTM, Boston Scientific, Cork, Ireland) advanced distally into the antero-medial branch. The embolic agent was prepared by mixing NBCA (Glubran 2, GEM; Viareggio, Italy) with the iodized oil (Lipiodol Ultra Fluid; Guerbet, Aulnay-sous-Bois, France) at a dilution of 1:5. After the injection of a few milliliters of 5% dextrose solution, small boluses (0.1–0.3 ml) of glue–oil mixture were continuously injected under fluoroscopic guidance using a blocked technique, for each side. In this technique, the microcatheter is advanced distally to nearly occlude the vessel, reducing intraluminal flow and facilitating controlled, targeted administration of the embolic agent. Injection was stopped when reflux occurred around the microcatheter involving the horizontal segment of the prostatic artery to obtain the entire artery occlusion up to its origin (Fig. 1). The procedure was suspended soon after the NBCA was attached to the vessel (about four seconds). Then, the microcatheter was quickly and firmly withdrawn, immediately cleaned by flushing it with dextrose and then passing a guidewire along its lumen. The same microcatheter was used on both pelvic sides. Technical success was defined as the embolization of at least one side of the prostatic gland with a clear visualization of the NBCA cast along the course and intra-prostatic branches of the embolized prostatic artery [17].

### Data collection

Baseline data that included patient age, comorbidities, medical therapy, IPSS, QoL, and PV were recorded. PV was assessed by dedicated software (3D-RA Rel.6, Philips Medical Systems; Amsterdam, the Netherlands) that generates a ‘real’ volume afterward measured on the MR. Follow-up data were collected to the time of data analysis, including changes in pharmacological therapy, IPSS, QoL, and PV. For patients with IUC, the interval between embolization and successful catheter removal was documented. Clinical outcomes (IPSS and QoL) and pharmacological therapy were analyzed at 12, 24, and 36 months, while PV was assessed only at 36 months. Any adverse events (AEs) were also registered according to the modified Clavien-Dindo grading system adapted to PAE as minor (I or II) or major (III, IV) [22].

For each procedure, fluoroscopy time (FT), air kerma, and dose area product (DAP) were automatically recorded by the angiographic system. These parameters were used to assess procedural time and radiation exposure. FT was defined as the total duration of fluoroscopic imaging, while air kerma and DAP quantified total exposure.

### Statistical analysis

Categorical variables were reported as absolute numbers and percentages; continuous variables as median and range (min–max). Fisher’s exact test was used for categorical comparisons, Mann–Whitney U for independent continuous variables, and Wilcoxon signed-rank test for paired data. A *p* value < 0.05 was considered significant. Analyses were performed using SPSS v27 (Windows) and GraphPad Prism v8.2.1 (macOS).

## Results

### Demographics and baseline characteristics

The study population consisted of 101 male patients with a median age of 79 years (range 64–89). Of these, 67 patients (66.3%; median age 78 years, range 64–87) presented with LUTS, while 34 patients (33.7%; median age 82 years, range 65–89) had an IUC at the time of the procedure, and all were included in the final analysis since no patients were excluded after enrollment. The median duration of catheterization prior to embolization was 10 months (range 2–23). Comparison of patient age between the LUTS and IUC groups revealed no significant difference (*p* = 0.069), indicating comparable age distribution across the cohorts. The patients with IUC were not included in the post-procedure evaluation of clinical outcomes and pharmacological therapy for BPH.

At baseline, prior to undergoing PAE, 7 patients (10.5%) were not receiving any pharmacological treatment for BPH. Twenty-one patients (31.3%) were on monotherapy, either with alpha-1 adrenergic antagonists or 5-alpha reductase inhibitors, while 39 patients (58.2%) were taking both medications in combination. Ten patients (9.9%) had previously undergone TURP, and among them, only one had an IUC at the time of embolization. Variables with numbers of men at risk at several time points are shown in Table 1.

**Table 1:** Numbers of men at risk

Months of follow-up	IPSS	QoL	PV
Before PAE	67	67	101
12	61	61	–
24	49	49	–
36	34	34	32

*PAE* = Prostatic artery embolization, *IPSS* = International Prostate Symptom Score, *QoL* = Quality of life, *PV* = Prostatic volume.

Baseline patient characteristics, including the use of anticoagulant therapy, which were present in 49.5% of the population (50/101 patients), are summarized in Table 2.

**Table 2:** Demographics and baseline characteristics

Comorbidity / Demographic	Value / N (%)
Age (LUTS group)	Median 78 yrs (range 64–87)
Age (CUR group)	Median 82 yrs (range 65–89)
At least one comorbidity	62 (61.4)
≥ 2 comorbidities	14 (13.9)
Atrial fibrillation	17 (17.4)
Arterial hypertension	14 (13.9%)
Myocardial infarction	9 (8.9)
Stroke	4 (4.0)
Transient ischemic attack	4 (4.0)
Diabetes mellitus	7 (6.9)
Chronic kidney disease	2 (2.0)
History of neoplastic disease	5 (5.0)
On anticoagulant therapy (DOACs or warfarin)	14 (13.9)
On antiplatelet therapy (aspirin or clopidogrel)	26 (25.7)
Both anticoagulant and antiplatelet	3 (3.0)
No anticoagulant/antiplatelet therapy	51 (50.5)

*LUTS* = Lower urinary tract symptoms,* IUC* = Indwelling urinary catheter*, DOACs* = Direct oral anticoagulants

### Technical outcomes and radiation exposure

PAE using NBCA was technically successful in 100% of cases, with embolization performed bilaterally in 94 (93%) patients (Fig. 1). Among the 101 patients treated, Type 3 vascular anatomy was identified in 4 cases (4.0%). As each patient underwent a single embolization session with no repeat procedures during the study period, radiation dose parameters were calculated per procedure and thus correspond to per patient values. Mean and median fluoroscopy times were 28.09 min (± 10.26) and 25.5 min (range 9–48), respectively. The mean and median air kerma were 896.7 mGy (± 496.7) and 849.7 mGy (range 30.88–2170), respectively. Mean and median dose area product (DAP) values were 270.2 Gy·cm^2^ (± 140.7) and 246.5 Gy·cm^2^ (range 10–548), respectively.

### Safety and tolerability

In our cohort of 101 patients, a total of 14 complications were observed, corresponding to an overall complication rate of 13.9%. All events were classified as minor and were managed conservatively, without the need for hospitalization or surgical intervention. The majority of complications were related to the presence of the urinary catheter placed before the procedure in patients with LUTS, accounting for 7 cases (6.9% of the total cohort). These included 5 episodes (5.0%) of acute urinary retention (AUR) resulting in delayed catheter removal, one case (1.0%) of urinary retention occurring two months after successful catheter removal at two weeks post-PAE, and one case (1.0%) of catheter malfunction due to prostatic tissue sloughing. These events involved exclusively the subgroup of 67 patients with LUTS, resulting in a complication rate of 10.4% within this group. Specifically, the incidence of AUR was 7.5%, and both late retention and catheter malfunction occurred in 1.5% of cases each. All these patients ultimately had their catheters successfully removed, with a median time to catheter-free status of 2 months (range 1.5–4). Inflammatory and infectious complications occurred in 7 patients (6.9%) across the entire study population. These included post-embolization syndrome in 3 patients (3.0%) and one case (1.0% each) of prostatitis, epididymo-orchitis, rectal edema with tenesmus, and penile pain. The overall complication rate was 5.9% (2/34) in the IUC group and 17.9% (12/67) in the LUTS group. However, comparison of adverse events between the LUTS and IUC groups revealed no significant difference (*p* = 0.138). All conditions were managed conservatively with anti-inflammatory drugs, antibiotics, and analgesics as needed. In the case of rectal symptoms, topical corticosteroid suppositories were administered, resulting in complete resolution within one week. The distribution of complications between groups is detailed in Table 3.

**Table 3:** Adverse event

Adverse event	N (%) Entire population	N (%) LUTS population	N (%) IUC population
Total complications	14 (13.9)	12 (17.9)	2 (5.9)
Urinary retention (AUC, early/late)	6 (5.9)	6 (9)	–
Catheter malfunction	1 (1.0)	1 (1.5)	–
Post-embolization syndrome	3 (3.0)	2 (3)	1 (2.9)
Prostatitis	1 (1.0)	1 (1.5)	–
Epididymo-orchitis	1 (1.0)	1 (1.5)	–
Rectal edema with tenesmus	1 (1.0)	–	1 (2.9)
Penile pain	1 (1.0)	1 (1.5)	–

*N* = Numbers of men who experienced an adverse event are expressed as a proportion of the study population*, LUTS* = Lower urinary tract symptoms*, IUC* = Indwelling urinary catheter*. AUR* = Acute urinary retention

### Clinical outcomes

**Fig. 2:**
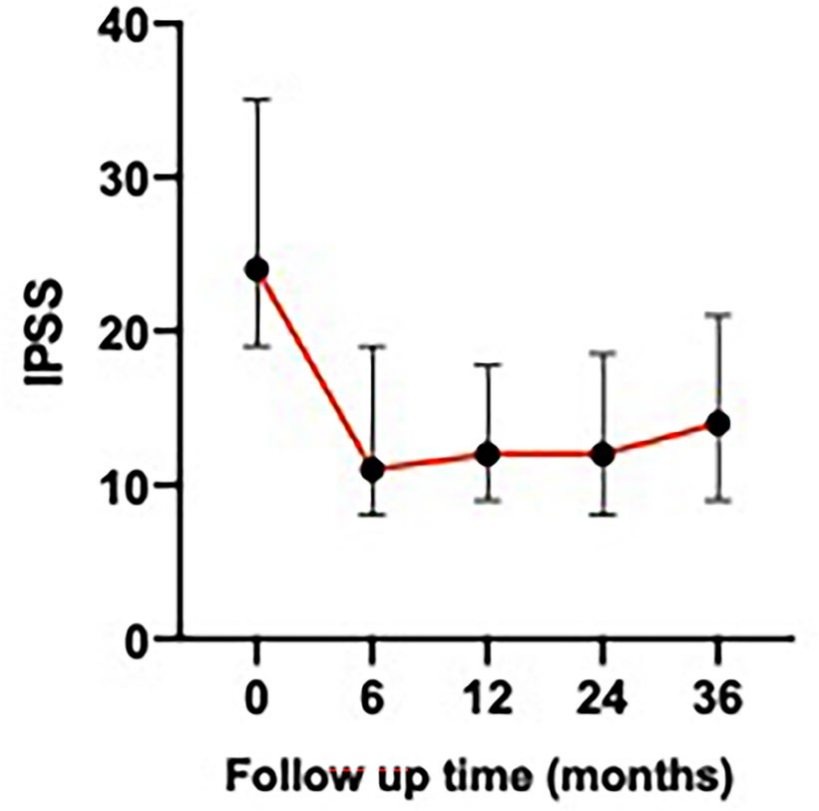
Line graph showing changes in the IPSS over time after PAE. Timepoint 0 corresponds to pre-PAE. Vertical bars represent point estimates and 95% confidence intervals

The clinical success rate was 93% (62/67 patients with LUTS) and the median follow-up was 26 months (range: 7–39 months). At baseline, the mean IPSS was 25.30 ± 8.13, with a median of 24.00 (range 8–35). A significant reduction was observed at 12 months (mean: 14.29 ± 7.94; median: 12.00 [range 2–35]) and 24 months (mean: 14.35 ± 8.38; median: 12.00 [range: 2–35]). At 36 months, a slight increase was noted (mean: 15.29 ± 7.81; median: 14.00 [range 4–35]), though values remained substantially lower than baseline. The overall change from baseline was statistically significant even at the 36-month follow-up (*p* < 0.001) (Fig. 2). Regarding QoL, the baseline mean score was 4.46 ± 1.12, with a median of 4.50. A marked reduction was observed at 12 months (mean: 2.16 ± 1.36; median: 2.00), which remained stable at 24 months (mean: 2.25 ± 1.39; median: 2.00) and 36 months (mean: 2.28 ± 1.35; median: 2.00). The QoL reduction from baseline was statistically significant (*p* < 0.001).

In patients with IUC, the clinical success was achieved in 25 of 34 cases (74%), with a median time to catheter removal of 29 days (range 23–55). Among the 9 patients in whom complete weaning was not achieved, 4 were transitioned to clean intermittent catheterization. The median follow-up was 29 months (range 10–43 months).

**Fig. 3:**
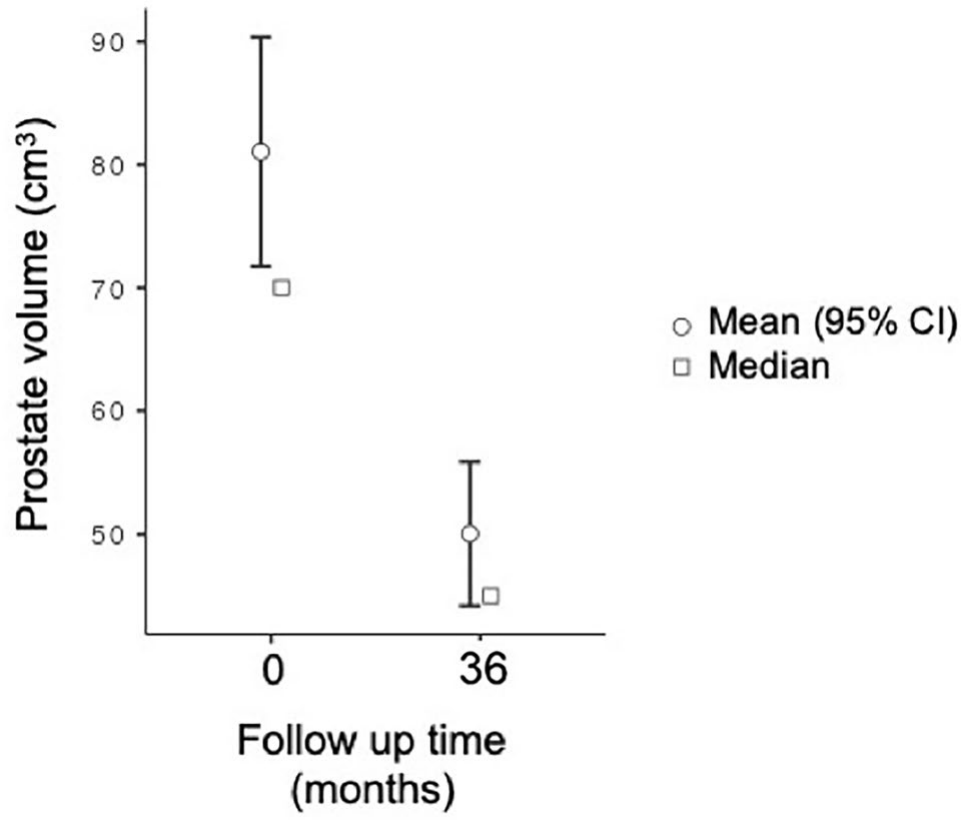
Changes in PV from baseline to 36 months after PAE. The graph shows both mean values with 95% confidence intervals (circles with error bars) and median values (squares). A sustained reduction in PV was observed over time

Regarding PV in the entire population, baseline measurements showed a mean volume of 80.14 ± 38.30 mL, with a median of 70 mL (range 40–260). At 36-month follow-up, the mean PV decreased to 50.04 ± 25.20 mL, with a median of 45 mL (range 19–160). This corresponds to mean and median volume reductions of approximately 37.6% and 35.7%, respectively (*p *< 0.001) (Fig. 3).

Pharmacological therapy for BPH and its changes during follow-up is summarized in Table 4. The overall changes from baseline were statistically significant (*p* < 0.05).

**Table 4:** Medication use before and after PAE

Medication regimen	Pre-PAE (N, %) N = 67	12 Months (N, %) N = 61	24 Months (N, %) N = 49	36 Months (N, %) N = 34
No medication	7 (10.5)	27 (44.3)	28 (57.1)	19 (55.9)
Alpha-1 adrenergic antagonists or 5-alpha reductase inhibitors	21 (31.3)	21 (34.4)	11 (22.5)	8 (23.5)
Alpha-1 adrenergic antagonists + 5-alpha reductase inhibitors	39 (58.2)	13 (21.3)	10 (20.4)	7 (20.6)

*PAE* = Prostatic artery embolization*. N* = Numbers of men with lower urinary tract symptoms pharmacological therapy

## Discussion

The results of our study confirm the feasibility, safety, and clinical efficacy of PAE performed with glue. In our series, the technical success rate is 100%, in line with previous studies using glue reporting success rates of 95–100% [16–18]. Our results are also in line with studies using microspheres, which reported technical success rates around 94–96% [2, 23]. From a clinical standpoint, our population demonstrates a significant and durable improvement in IPSS, with a 43% reduction in mean IPSS at 12 months, 44% at 24 months, and 40% at 36 months. These improvements are comparable to those reported in NBCA-based series but with short follow-up: Loffroy et al. [17] documented a 56% reduction in IPSS at 6 months, while Bamshad et al. [18] observed a mean IPSS decrease of 11 points at 7 weeks. Our QoL improvement (mean decrease of > 2 points sustained over time) also mirrors outcomes from glue series (mean reduction of 2.8–3 points) and aligns with the improvements reported in microsphere-based studies, such as the two-year PROstate study (QoL decrease from 4.7 to 2.3) [23].

The PV reduction in our cohort is substantial, with a mean decrease of 37.6% and a median of 35.7% at 36 months. These results align with previous NBCA-based studies reporting 20–33% reductions at 3–6 months [16–18] and are comparable to the ~ 39% reduction observed with microspheres [2].

In patients undergoing PAE for IUC, resuming spontaneous micturition is a key clinical goal. In our cohort, 74% regain spontaneous voiding within a median of one month, with 71.4% catheter-free at 36 months. These results are comparable to the PROstate study by Sapoval et al. [23], which reported catheter removal in 65.8% within 90 days and sustained catheter-free status at 24 months. Similarly, Pouchot et al. [24] reported 80.7% catheter removal within two months and 79% catheter-free status at two years.

An important aspect of our study population is the advanced age of the treated patients, both in the LUTS (mean age of 78 years) and IUC (mean age of 82 years) groups. These values are consistently higher than those reported in the majority of published studies. For instance, the mean age in the PROstate study was 69 years in the LUTS cohort and 75 in the IUC cohort [23]; the median age was 69 years in the NBCA-based studies by Loffroy et al. [-16–17], and 68.3 years in the comparative study by Salet et al. [14]. Our experience therefore represents a very elderly male cohort, reflecting the ongoing demographic shift toward an aging population in Western countries. In Italy, for example, the average male life expectancy is 81.4 years and 81.6 years in the region where our institution is located [25]. This observation has both clinical and epidemiological relevance. Elderly patients often present with multiple comorbidities and limited suitability for surgery, highlighting the clinical value of minimally invasive approaches such as PAE. Moreover, the demonstrated efficacy and safety of PAE in our old-old patients population (≥ 75 years) support its use even in advanced age, aligning with the growing need for age-adapted, minimally invasive therapies for BPH in contemporary urological practice. In this context, reducing or eliminating pharmacologic therapy for BPH is particularly relevant, given that elderly patients are frequently polymedicated; notably, 62% of our cohort had at least one comorbidity. Following PAE, the percentage of patients not receiving any BPH-related medication increased from 10.5% at baseline to 55.9%, while those on combination therapy decreased from 58.2% to 20.6% at 36 months. The results of the present study highlight the potential of PAE in this patient population, to reduce medication burden, improving overall treatment adherence and minimizing side effects.

One key procedural advantage confirmed in our experience is reduced FT. In our cohort, median FT was 25.5 min, comparable to other NBCA-based studies. Bamshad et al. [18] reported a median FT of 22.2 min, while Sanghvi et al. [15] found significantly shorter FT with NBCA vs. microspheres (19.8 vs. 30 min, *p* < 0.001). Similarly, Salet et al. [14] reported shorter FT with glue (21.4 vs. 33.3 min, *p* < 0.01). In contrast, FT with microspheres typically ranges from 30 to 40 min [2, 23]. NBCA enables faster embolization with a single, well-positioned injection and improved visibility due to the glue–oil radiopacity. This procedural efficiency is especially relevant in elderly or frail patients, reducing discomfort and immobilization time, and reflects a broader trend in interventional radiology toward minimizing procedure duration [26, 27]. Moreover, it shortens overall procedure duration compared with conventional PAE, historically characterized by long interventions, difficult catheterization, and a steep learning curve. Such efficiency also has implications for cost-effectiveness, by limiting resource use (hospital stay, anesthesiologic support, operating room time) and potentially reducing reliance on chronic medication, in line with sustainable healthcare principles [28].

Despite the lower FT, radiation dose (DAP and air kerma) did not show a proportional reduction. This aligns with findings from Sanghvi et al. [15], who found no significant differences between glue and microsphere groups. In our workflow, CBCT is routinely performed and contributes substantially to cumulative exposure, even though it is not included in fluoroscopy time. Additional DSA acquisitions further increase the overall dose. Thus, shorter procedures do not always mean lower radiation. Future strategies for dose optimization may include technological advances such as artificial intelligence, which could improve image interpretation and procedural planning, thereby reducing reliance on CBCT and DSA in selected cases [29, 30].

Our findings confirm the overall safety and tolerability of PAE using glue, with a complication rate of 13.9%, all of which were minor (modified Clavien-Dindo ≤ II) and managed conservatively. These results are in line with published NBCA-based studies, which report minor adverse event rates ranging from 18 to 23%, and no major complications [14, 15, 18]. Likewise, particle-based PAE demonstrates a low complication profile, suggesting that both embolic agents are comparably safe [2, 23].

A distinctive feature of our cohort was the 10.4% incidence of post-procedural urinary retention, observed in patients who underwent prophylactic urinary catheter placement. This management strategy was inherited from our initial experience with microparticles in order to facilitate prostate localization, prevent discomfort during long procedures, and minimize the risk of post-ischemic AUR. Interestingly, this approach differs from other published NBCA series, in which no urinary catheterization was routinely performed, and no cases of AUR were reported [14, 16–18]. The higher rate of AUR in our series may be partially explained by the advanced age of our patient population, many of whom likely exhibit pre-existing bladder dysfunction or reduced detrusor contractility. In this context, the re-establishment of spontaneous voiding post-procedure may be delayed or complicated. These observations suggest that pre-procedural catheterization should not be standardized but rather individualized based on patient characteristics, especially in older patients with borderline bladder function or limited voiding reserve.

This study has some limitations. It is a retrospective, single-center analysis, which may introduce selection bias. Our findings were derived from an older patient population; thus, their applicability to younger patients remains uncertain, particularly concerning long-term efficacy. Follow-up duration was variable and, although relatively long compared to other NBCA studies, remains heterogeneous among patients, with some cases having less than 12 months of data, potentially underestimating late recurrences or delayed complications. Functional urodynamic parameters, such as Q_max_ and post-void residual volume, were not systematically collected, limiting the evaluation of objective functional improvement. This choice was deliberate, as previous studies consistently show inferior urodynamic outcomes for PAE compared with TURP; instead, we focused on long-term clinical outcomes such as IPSS, QoL, PV reduction measured on imaging, and catheter removal.

Our routine use of pre-procedural urinary catheterization may have influenced the observed rate of post-procedural AUR. Finally, the systematic use of CBCT, while helpful for anatomical assessment, contributed to radiation exposure and may not reflect standard practice in other institutions. Moreover, the absence of a control group limits direct comparisons, although our findings are consistent with the literature, and a TURP control group was not feasible in our elderly population unfit for surgery.

This is the first study to report long-term outcomes of PAE using glue, confirming its safety and sustained efficacy. The procedure was especially beneficial in elderly patients, often burdened by comorbidities and poor surgical candidacy, offering a valuable minimally invasive alternative. Further prospective, multicenter studies with standardized protocols and control groups are needed to validate these findings and refine patient selection.
