# Correction: Prostatic artery embolization with glue for benign prostatic hyperplasia in elderly patients: three-year results

**DOI:** 10.1007/s11547-025-02162-0

**Published:** 2025-12-08

**Authors:** Antonio Vizzuso, Maria Vittoria Bazzocchi, Mara Bacchiani, Giorgia Musacchia, Antonio Spina, Eugenia Fragalà, Giovanna Venturi, Enrico Petrella, Roberta Gunelli, Emanuela Giampalma, Matteo Renzulli

**Affiliations:** 1 Radiology Unit, Morgagni-Pierantoni Hospital, AUSL Romagna, 47121 Forlì, Italy; 2 Department of Urology, University of Florence, Florence, Italy; 3 Urology Unit, Morgagni-Pierantoni Hospital, AUSL Romagna, 47121 Forlì, Italy; 4 Medical Physics and Clinical Engineering Operative Unit, Morgagni-Pierantoni Hospital, AUSL Romagna, 47121 Forlì, Italy; 5 Department of Medical and Surgical Sciences, University of Bologna, 40100 Bologna, Italy

**Correction to: La radiologia medica** 10.1007/s11547-025-02136-2

In this article, author would like to correct one of the author name in the published version as given below:

Published version: Renulli

Should be corrected to: Renzulli

The Original article has been corrected.

